# Psychometric properties of a Norwegian adaption of the Barratt Impulsiveness Scale‐11 in a sample of Parkinson patients, headache patients, and controls

**DOI:** 10.1002/brb3.605

**Published:** 2016-11-23

**Authors:** Jonas C. Lindstrøm, Nora G. Wyller, Marianne M. Halvorsen, Silje Hartberg, Christofer Lundqvist

**Affiliations:** ^1^ Health Services Research Centre (HØKH)Akershus University HospitalLørenskogNorway; ^2^ Institute of Clinical MedicineCampus Akershus University HospitalUniversity of OsloOsloNorway; ^3^ Department of NeurologyAkershus University HospitalLørenskogNorway

**Keywords:** Barratt Impulsiveness Scale, headache, impulsivity, Parkinson's disease, reliability, validity

## Abstract

**Objective:**

To assess the psychometric properties of a Norwegian translation of the Barratt Impulsiveness Scale (BIS‐11) for use in populations of headache, Parkinson's disease (PD), and healthy controls.

**Materials and Methods:**

The BIS‐11 was forward and backward translated by native speakers of both Norwegian and English to give Norwegian BIS‐11 (Nor‐BIS‐11). A convenience sample (110 subjects) of healthy controls (47), PD patients (43), and chronic headache patients (20) (the latter two recruited from a Neurology outpatient clinic), were asked to complete the scale (a subset twice for test–retest). Exploratory and confirmatory factor analyses were done for a single‐factor model, the original three‐factor model and a two‐factor model. Test–retest results were analyzed using the Bland–Altman approach.

**Results:**

The Nor‐BIS‐11 scale showed good utility and acceptability as well as good test–retest reliability in this sample. Cronbach's α was .68, test–retest bias was −0.73, Cohen's δ = −.134, and limits of agreement were −11.48 to 10.01. The factor structure was found to fit better with a two‐factor model than with the original model with three factors. The model fit indices indicated a moderate fit.

**Conclusions:**

The Nor‐BIS‐11 scale is acceptable and reliable to use in Parkinson's disease patients, chronic headache patients, and healthy controls. The results should be interpreted in a two‐factor model but with caution due to low construct validity. External validity needs to be further tested.

## 1 Introduction

There has, over the past few decades, been an increasing focus on impulsivity as an important component of several disease states both within psychiatry and somatic illness. This includes depression and anxiety, personality disorders, ethanol‐ and drug‐associated behavior, Parkinson's disease (PD), headache disorders, and disorders related to brain damage and dementia. Impulsivity is also very variable as a personality determinant within control populations including both younger individuals and elderly.

An Impulse Control Disorder (ICD) is defined as “the failure to resist an impulse, drive, or temptation to perform an act that is harmful to the person or to others” (DSM‐IV).

There have been many studies focusing on ICDs and impulsive behavior among young adults and youth (Cosi, Vigil‐Colet, Canals, & Lorenzo‐Seva, 2008; Hartmann, Rief, & Hilbert, 2011; Li & Chen, 2007; von Diemen, Szobot, Kessler, & Pechansky, 2007). Less is known of impulse control among elderly. Recently, there has been more focus on ICDs and impulsive behavior among PD patients on dopaminergic medication. ICDs can have potentially devastating consequences for both patient and the patient's family (Voon et al., 2006). The direct interaction of dopaminergic medication as well as the demonstrated prevalence of compulsive medication behavior in PD (Callesen, Scheel‐Kruger, Kringelbach, & Moller, 2013; Giovannoni, O'Sullivan, Turner, Manson, & Lees, 2000 for review), is of theoretical interest also considering studies on involvement of dopaminergic mechanisms in addictive behavior (Volkow & Morales, 2015). Compulsive, addiction‐like, behavior has been suggested to be involved in the transformation of frequent headaches into the form of chronic headache designated “medication‐overuse headache” (Calabresi & Cupini, 2005). Indeed, both using functional imaging and psychometric tests, plausible involvement of impulse‐controlling and decision‐making pathways have been suggested (Biagianti et al., 2012; Ferraro et al., 2012). We have previously demonstrated that applying behavioral treatment originally adapted for treatment of addiction of alcohol and illegal drugs, the chronic headache of individuals with medication‐overuse headache may be reversed, thus strengthening the notion that impulse control systems are involved (Kristoffersen et al., 2015). In both PD and addictive disorders, including medication overuse in chronic headache, many other scales which measure disease‐specific impulsive behavior have been used such as the Questionnaire for Impulsive‐compulsive disorders in PD (QUIP), the Severity of Dependence Score (SDS), and others (Gossop et al., 1995; Lundqvist, Aaseth, Grande, Saltyte‐Benth, & Russell, 2010; Weintraub, Papay, & Siderowf, 2013). It may, however, be of interest to compare impulsive behavior between different conditions using a general scale. The inappropriateness of certain disease‐specific questions in other disease settings, may also suggest that a general scale for behavioral impulsiveness may have advantages.

The Barratt Impulsiveness Scale (BIS‐11) is a 30‐item self‐report questionnaire assessing the personality/behavior construct of impulsiveness (Stanford et al., 2009), originally developed by Barratt (1959) to analyze the relationship between anxiety and impulsiveness. It was constructed to measure impulsivity as a unidimensional personality trait, but was later changed and developed to include several dimensions (Patton, Stanford, & Barratt, 1995). The current Barratt scale, BIS‐11, proposes that impulsivity is a construct of three broad dimensions: motor, nonplanning, and attentional impulsiveness. The BIS‐11 has been used to assess impulsivity across a variety of populations and external validity has been extensively tested against clinical diagnoses such as substance use disorders, mood disorders, attention‐deficit hyperactivity disorders as well as aggressive and violent behavior in criminal populations. BIS‐11 has furthermore been correlated against punishment and reward sensitivity, attention, and cognitive function including learning and decision‐making (Stanford et al., 2009). The scale asks about the frequency of impulsivity‐related behaviors and each item is scored on a 4‐point scale. The higher the summed score for all items, the higher the level of impulsiveness (Patton et al., 1995). The BIS in its 11th revision (Patton et al., 1995) is now the most commonly administered self‐reported measure specifically designed to assess impulsiveness (Stanford et al., 2009). Stanford et al. (2009) suggest that the BIS‐11 should be viewed as a standard point of reference in research on impulsiveness. The scale has begun to be used in studies of impulsivity among samples of Parkinson patients (Antonini et al., 2011) but has, to the best of our knowledge not been used in medication‐overuse headache or other chronic headaches.

The aim of this study was to develop and internally validate a Norwegian translation of the BIS‐11 scale in a mixed convenience population of clinical neurological cases (PD patients and patients with chronic headache) and controls.

## 2 Materials and Methods

### 2.1 Participants and recruitment

Subjects were a convenience sample of 110 patients from a neurological outpatient clinic as well as healthy controls recruited among hospital staff in a Norwegian Hospital. Only basic demographic information (age and gender) as well as the tentative diagnosis was obtained.

Participants were asked to complete the questionnaire themselves, but could get some assistance by the research nurse if necessary. The questionnaire was completed prior to the appointment to the relevant physician in the outpatient department. For test–retest, the questionnaire was completed again approximately 1 hr later by 27 patients.

### 2.2 Translation

The 30‐item BIS‐11 scale was translated into Norwegian by volunteers from a broad range of professional backgrounds, all fluent in English. Five people translated the BIS‐11 scale from English into Norwegian. Two people with background in psychology and medicine checked the convergence of the five Norwegian versions with reference to content and wording. Further, two people with solid knowledge in English and psychology then back‐translated the scales into the original language.

### 2.3 Factor structure

In addition to the total score of all 30 items, we also investigated different subscales and whether our sample conformed to underlying factor structures published elsewhere.

#### 2.3.1 Three‐factor model

The original structure using the three‐second‐order factors was tested (Patton et al., 1995). The three factors are attentional (items 5, 6, 9, 11, 20, 24, 26, and 28), motor (items 2, 3, 4, 16, 17, 19, 21, 22, 23, 25, and 30), and nonplanning (items 1, 7, 8, 10, 12, 13, 14, 15, 18, 27, and 29) impulsiveness. Each of these three factors were described as being composed of two underlying factors each, giving a total of six underlying factors. However, the six‐factor structure was not analyzed here.

#### 2.3.2 Two‐factor model

Reise, Moore, Sabb, Brown, and London (2013) found a two‐factor model to be more optimal, thus we decided to analyze also this structure. The two factors suggested are cognitive impulsivity (items 1, 2, 5, 7, 8, 9, 10, 12, 13, 14, 15, 18, 20, 22, 23, 25, 27, 29, and 30) and behavioral impulsivity (items 2, 5, 6, 10, 11, 14, 16, 17, 19, 21, 22, 24, 25, 26, 28). Items 3 and 4 of the original scale were removed and items 2, 5, 10, 14, 22, and 25 belong to both factors.

### 2.4 Statistics

Mean and standard deviations of the scores in the complete sample as well as in each patient sample are reported for the total and the three subscales. In addition, we present the scores on the two subscales implied by the factor structure found by Reise et al. (2013). ANOVA is used to test for differences between the three groups on the different subscales.

To assess the test–retest reliability, we used the Bland–Altman approach (Bland & Altman, 1999). For each subject, the test–retest difference in total score was plotted against the mean of the difference. The average differences (bias) and limits of agreement were calculated. In addition, we calculated Pearson correlation coefficients between repeated measurements.

Exploratory factor analyses were done using principal component analysis and Promax rotation for both a three‐factor model and a two‐factor model. Confirmatory factor analyses were also performed using the predecided three‐factor structure of Patton et al. (1995) and the two‐factor structure of Reise et al. (2013). The two‐factor model was fitted with the loadings for item 3 and 4 set to zero for both factors in order to have comparable Akaike information criterion (AIC) values to the one‐ and three‐factor models. A model was also estimated where these two items were completely removed.

For these analyses, the values of the AIC, χ^2^, comparative fit index (CFI), and the Tucker‐Lewis index are given. Full information likelihood estimation was used to handle missing values.

All statistical analysis was done using R (R Core Team, 2015). The psych package was used for exploratory factor analysis and calculating Cronbach's α (Revelle, 2015). The lavaan package was used for confirmatory factor analysis (Rosseel, 2012).

### 2.5 Ethical issues

All participants provided written informed consent and the study was approved by the Regional Committee for Medical and Health Research Ethics in South‐Eastern Norway and the data inspectorate officer of the hospital.

## 3 Results

### 3.1 Population and observational notes

The sample included a total of 20 chronic headache patients (16 females, 4 males, mean age: 41.1, age range: 20–52), 43 PD patients (13 females, 30 males, mean age: 69.5, age range: 42–85), and 47 healthy controls (38 females, 9 males, mean age: 37.6, age range: 21–63), that is, all 110 participants were included. Twenty‐seven participants (seven chronic headache, 12 PD, and eight healthy controls) completed the questionnaire twice for test–retest.

Participants, both patients and healthy controls, generally had no problem completing the questionnaire and did not feel provoked by the questions. Parkinson patients had some more missing items motivated by some questions not being felt as being applicable—for example, “I plan for job security” and “I change jobs” were sometimes left out by patients on pension or on sick leave as not being applicable.

### 3.2 Missing values

One PD patient answered only 16 items and was excluded from some of the analyses. Nine participants left one item unanswered and six participants did not answer two items. In addition, four other participants did not answer between three and five items. The item with most missing answers was item 16 (“I change jobs”), which eight subjects (7.3%) did not answer. Seven of these were PD patients. The other items were left unanswered by at most three participants (2.8%).

### 3.3 Internal consistency and reliability

**Figure 1:**
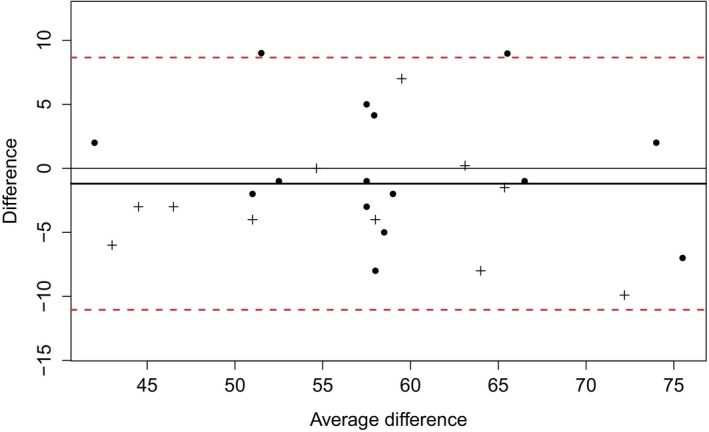
Bland–Altman plot of test–retest variability. The difference between the test and retest is plotted against the mean of the difference for 26 participants (one excluded). Parkinson's disease patients are indicated with a + sign. The mean of the differences is shown as a bold black line and is close to 0, indicating low retest bias. The two dashed red lines are the 95% limits of test–retest agreement, which are approximately +/− 10 points, indicating moderate variability

Cronbach's α was calculated based on all pairwise complete observations. Cronbach's α was .68 for the complete sample indicating a close to acceptable internal consistency. It was lowest for the control group (0.60), while it was acceptable (0.70) for Parkinson's patients and chronic headache patients (0.76). Test–retest reliability (Pearson correlations, *r*) for the total score as well as for both the two‐factor and the three‐factor models was greater than 0.7 in all diagnosis groups, except for total score (*r* = .65), cognitive (*r* = .64), and nonplanning (*r* = .06) among the healthy controls. The Bland–Altman plot is shown in Figure 1. Test–retest bias was −0.73 (95% CI −2.90 to 1.43, Cohens δ = −0.134) and limits of agreement were −11.48 to 10.01.

### 3.4 Factor analyses

#### 3.4.1 One‐factor model

In the confirmatory factor analysis, the one‐factor “total score” model gave fit indices as shown in Table 1 indicating the poorest fit to the data of the factor structures tested.

**Table 1:** Fit indices of confirmatory factor analyses for three different factorial models for Barratt Impulsiveness Scale‐11

Model	χ^2^ (*df*)	AIC	TLI^a^	CFI^b^
One Factor	785.9 (405)	7655.8	0.215	0.269
Two Factor (loadings for item 3 and 4 set to 0)	686.4 (399)	7568.4	0.399	0.448
Two Factor (item 3 and 4 removed)^c^	–	–	0.427	0.480
Three Factor	704.2 (402)	7580.2	0.373	0.420

a Tucker‐Lewis index.

b Comparative fit index.

c χ^2^ and Akaike information criterion (AIC) not reported as these are not comparable to the rest of the models.

#### 3.4.2 Two‐factor model

**Figure 2:**
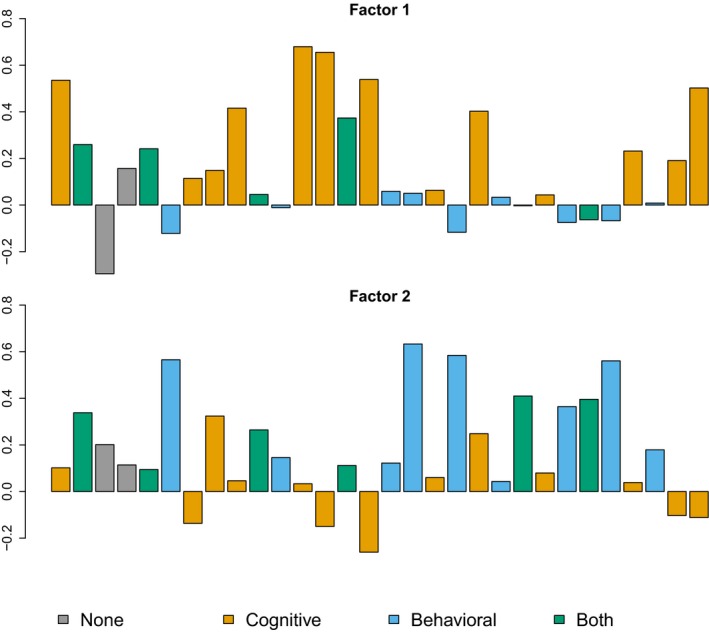
Exploratory factor analysis, two‐factor model. Barplot of loadings for items 1–30 for the Barratt Impulsiveness Scale‐11 two‐factor model

The loadings from the exploratory factor analysis for a two‐factor model are given in Figure 2. The two factors fit well with the “cognitive” versus the “behavioral” factors suggested by Reise et al. (2013). Of items suggested by Reise to load on both factors, we found that items 10, 22, and 25 loaded almost exclusively on factor 2 (close to the “behavioral” factor), while items 2, 5, and 14 also in our case had large loadings on both factors. The results of confirmatory factor analyses of this model are presented in Table 1, both the model with loadings for item 3 and 4 set to 0, and where these were completely removed from the analysis. The standardized item loadings for the two‐factor model are also given in Table S1.

#### 3.4.3 Three‐factor model

**Figure 3:**
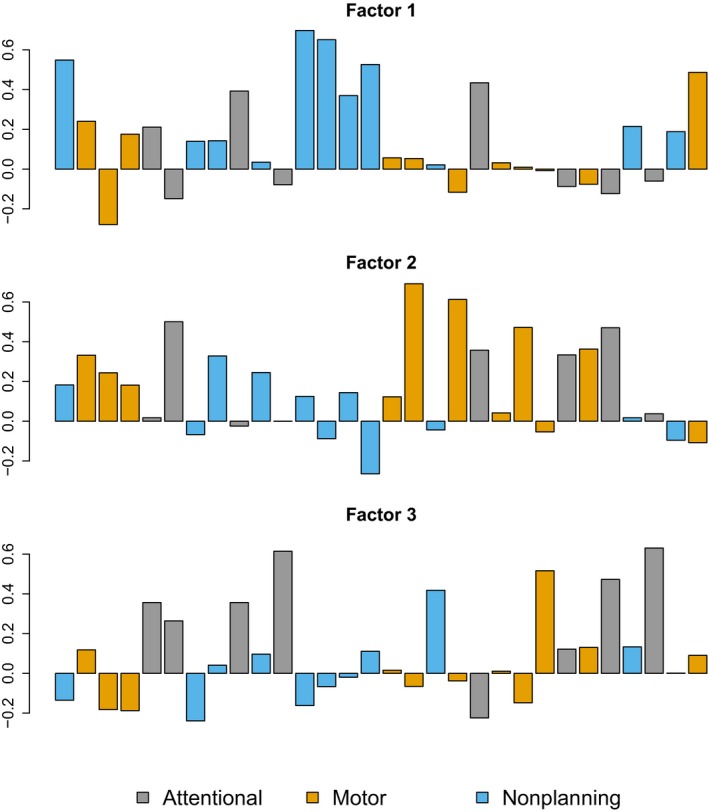
Exploratory factor analysis, three‐factor model. Barplot of item loadings for items 1–30 for the Barratt Impulsiveness Scale‐11 three‐factor model

The loadings from an exploratory factor analysis for three factors are given in Figure 3. Observationally, none of the factors seemed to map the original three‐second‐order factors (Patton et al., 1995). The originally described “nonplanning” factor seemed to map mainly to factor 1 in our material, while the “motor” factor mainly consisted of items in factor 2, here with the “attentional” factor consisting of a mix of all three‐factor groups (Figure 3). The results of confirmatory factor analyses of this model are presented in Table 1.

#### 3.4.4 Comparison between different factor structures

The results of the confirmatory factor analyses are given in Table 1. The two‐factor model had lowest AIC and showed greatest fit using the TLI and CFI fit indices, thus showing the best fit to our data. The one‐factor model had the poorest fit to the data. The differences between each of the factor models are significant (χ^2^‐test, *p* < .001).

### 3.5 Age and diagnosis

Linear regression analysis showed no significant association between age and BIS‐11 scores (Table 2). This was true for total scores and the subscales implied in the two‐factor model and the three‐factor model. The results suggested a different relationship with age for PD patients compared to controls and chronic headache patients. As PD patients were also notably older, we also performed the same analyses excluding the PD patients. This showed a nominal significant relationship (*p* = .049) between age and motor impulsivity scores in the three‐factor model as well as a nonsignificant tendency (*p* = .071) for a similar association between age and behavioral impulsivity in the two‐factor model. Increasing age gave lower impulsiveness scores.

**Table 2:** Linear regression analyses of relationship between Barratt Impulsiveness Scale‐11 score and age with and without Parkinson's disease (PD) patients

Subscale	Incl. PD patients		Excl. PD patients	
	Regression coefficient (change per year)	*p*‐value	Regression coefficient (change per year)	*p*‐value
Total	.026	.54	−.048	.56
Attention	.033	.07	−.027	.46
Motor	−.022	.17	−.067	.049
Nonplanning	.014	.51	.046	.31
Cognitive	.024	.47	.045	.50
Behavioral	−.008	.75	−.090	.07

The score on the total scale and subscales are shown in Table 3. There were no significant differences between the three diagnosis groups regarding total score or score for the different subscales.

**Table 3:** Mean (standard deviation) for Barratt Impulsiveness Scale‐11 score and subscales

Subscale	All (*n *=* *110)	Healthy (*n *=* *47)	PD (*n *=* *43)	Headache (*n *=* *20)	*p*‐value
Attention	15.16 (3.43)	14.34 (2.88)	16.01 (3.92)	15.29 (3.18)	.07
Motor	19.71 (3.09)	19.84 (2.74)	19.61 (3.44)	19.63 (3.23)	.93
Nonplanning	24.45 (4.13)	23.92 (3.41)	24.52 (4.59)	25.57 (4.58)	.32
Cognitive	39.49 (6.16)	38.52 (5.05)	39.93 (6.92)	40.79 (6.74)	.32
Behavior	25.85 (4.61)	25.64 (4.14)	25.99 (5.23)	26.06 (4.45)	.92
Total	59.37 (7.89)	58.12 (6.10)	60.20 (9.25)	60.49 (8.45)	.36

As a sensitivity analysis, the analyses done with the patient only answering 14 items were excluded. The results did not differ substantially.

## 4 Discussion

The BIS‐11 scale translated into Norwegian showed good utility and acceptability as well as good test–retest reliability in a sample of PD patients, chronic headache patients, and healthy controls. The factor structure was analyzed and found to fit better with a two‐factor model encompassing the cognitive and behavioral factors described by Reise et al. (2013) than with the original model with three‐second order factors (Patton et al., 1995). The model fit indices indicated a moderate fit at best.

The population participating in this study was a convenience sample of chronic headache patients and Parkinson patients from a neurological outpatient clinic as well as separately recruited healthy controls (largely from hospital staff). Controls were age and gender matched against the headache group and therefore considerably younger than the PD patients. Here, we have only validated the Norwegian BIS‐11 for use in these patient groups. However, given the widespread use of the BIS‐11 in international literature and the validation presented here, we suggest that, providing the use of relevant control groups and the factor structure presented here, results achieved using the Norwegian BIS‐11 should be valid.

Our test–retest interval (1 hr) may be too short to avoid direct recall. However, many of the included PD patients had advanced fluctuating Parkinson and in view of recent reports of fluctuating nonmotor symptoms (Fauser et al., 2015), we chose a time interval short enough to avoid these short‐term fluctuations. Assessment was made before and after an outpatient consultation, thus enabling us to see if patients were in a similar on–off state at both completions of the BIS‐11. The participants were not told in advance that they would have to complete the questionnaire one more time. The second time all were told that they should answer the questions anew and not try to remember what they had answered the last time. Previous studies have used varying test‐recall test times of 2 to 6 months (Fossati, Di Ceglie, Acquarini, & Barratt, 2001; Gülec et al., 2008; Hartmann et al., 2011; Someya et al., 2001). Though our test–retest correlations were high, they were generally at a similar level as these studies except for some of the factors in the three‐factor models from some studies, which had a reduced test–retest correlation, the lowest (*r* = .30 and *r* = .37 for nonplanning and motor impulsivity, respectively), as expected, being for the study with the longest test–retest interval (Hartmann et al., 2011).

The BIS‐11 is the most commonly used impulsivity instrument both across age groups, case groups, and languages. The original scale devised by Barratt in 1959 has been revised several times (Barratt, 1959). The originally suggested factor structure of the present version, the BIS‐11 was described by Patton et al. (1995) who modified the original a priori three‐factor structure based on an exploratory principal component analysis which verified the second‐order factors “motor impulsiveness” and “nonplanning impulsiveness”, but could not verify a “cognitive impulsiveness” factor. Rather, the factor termed “attentional impulsiveness” was suggested (Patton et al., 1995). This three‐factor model has since, to a large extent been followed in several validation articles of different language versions of the BIS‐11 (Spanish: [Cosi et al., 2008]; Portuguese: [von Diemen et al., 2007]; Turkish: [Gülec et al., 2008]; Chinese: (Li & Chen, 2007; Lu, Jia, Xu, Dai, & Qin, 2012]; Italian: [Fossati et al., 2001]). However, it is noticeable that the factor structure differs considerably between the different BIS‐11 versions. In line with this, Reise et al. (2013) proposed a new two‐factor structure reflecting cognitive and behavioral impulsivity. In the present paper, we also found evidence that a bifactor structure was a superior model than both the original model with three‐second order factors and a one‐factor total model. Reise et al. (2013) described factor loadings (for a total of 11 parcels in the bifactor model) of 0.28 to 0.76 for parcels associated with factor one (cognitive impulsivity) and 0.24 to 0.80 for factor two parcels (behavioral impulsivity). The lowest loadings for both were for parcels describing “buying and spending sprees” (0.24 and 0.28) and “no cognitive mediation” (0.37 and 0.39) which belonged to both factors. These authors also suggested that an 11‐item “brief‐BIS” scale could be used if a unidimensional score for impulsivity is desired rather than a one‐factor interpretation of the full BIS‐11 (see also Steinberg, Sharp, Stanford, & Tharp, 2013) and found no support that interpreting the BIS‐11 score as based on three subscales structure gave meaningful mapping to psychological constructs (Reise et al., 2013). The relationship between BIS‐11 factors and psychological laboratory tests of impulsivity as well as axis I or axis II diagnoses also show that different factors map to different psychological aspects (Swann, Bjork, Moeller, & Dougherty, 2002).

The BIS‐11 scale has been used in several different settings where impulsivity may be important. It has been validated for use in children/adolescents (Cosi et al., 2008; Hartmann et al., 2011; Li & Chen, 2007; von Diemen et al., 2007) and among elderly (Tamam, Bican, & Keskin, 2014). BIS‐11 has also been used among PD patients Antonini et al., 2011; Smulders et al., 2014 among patients with depression (Lu et al., 2012; reviewed in Saddichha & Schuetz, 2014) as well as in combinations of both (Fonoff et al., 2015). ICDs have also been studied (binge eating: [Akkermann et al., 2010], substance dependence: [Gülec et al., 2008], prison inmates: [Patton et al., 1995], elderly with ICDs: [Tamam et al., 2014]). Various language versions have also been well validated in control subjects (Fossati et al., 2001; Gülec et al., 2008; Hartmann et al., 2011; Li & Chen, 2007; Patton et al., 1995; Reise et al., 2013; Someya et al., 2001; Steinberg et al., 2013).

We have here validated the Norwegian translation in three samples: healthy (young) controls, chronic headache patients, and PD patients, the latter being of an older age. We suggest that the scale can be used in these settings providing caution in the interpretation and, in the mapping to theoretical constructs. We suggest the Norwegian translation may be interpreted best using the bifactor model described by Reise et al. (2013). If a total score is required, the brief‐BIS score may possibly be used though this was not tested here, neither have we tested external validity versus other measures as this is conceivably situation and sample dependent.

## 5 Conclusion

Our version of the BIS‐11 scale is acceptable and reliable to use in our study population including PD patients, chronic headache patients, and healthy controls. The results should be interpreted with caution due to moderate construct validity. We suggest results should be presented both as total score and as two separate factors describing cognitive and behavioral impulsivity, respectively. External validity needs to be further tested.

## Conflict of Interests

The authors declare no other conflicts of interests.
