# Supplementary material for: Psychometric properties of a Norwegian adaption of the Barratt Impulsiveness Scale‐11 in a sample of Parkinson patients, headache patients, and controls
Source: Brain Behav. 2016 Nov 23;7(1):e00605. doi: 10.1002/brb3.605 (PMC5256188; doi:10.1002/brb3.605)
Supplement: Supplementary file 1 [file BRB3-7-e00605-s001.docx]

***Supplemental table 1*** Factor loadings from confirmatory factor analysis of two factors.

| Item # | Behavioural | Cognitive |
| --- | --- | --- |
| 1 |  | 1.000* |
| 2 | 0.531 | 0.277 |
| 5 | 0.139 | 0.276 |
| 6 | 0.863 |  |
| 7 |  | 0.218 |
| 8 |  | 0.234 |
| 9 |  | 0.595 |
| 10 | 0.671 | 0.033 |
| 11 | 0.291 |  |
| 12 |  | 0.979 |
| 13 |  | 1.157 |
| 14 | 0.209 | 0.475 |
| 15 |  | 0.810 |
| 16 | 0.089 |  |
| 17 | 1.108 |  |
| 18 |  | 0.072 |
| 19 | 1.000* |  |
| 20 |  | 0.731 |
| 21 | 0.064 |  |
| 22 | 0.782 | 0.014 |
| 23 |  | -0.020 |
| 24 | 0.459 |  |
| 25 | 0.518 | -0.075 |
| 26 | 0.874 |  |
| 27 |  | 0.296 |
| 28 | 0.382 |  |
| 29 |  | 0.182 |
| 30 |  | 0.587 |
|  |  |  |
|  |  |  |
| *fixed to 1. | |  |
